# The value of different CT‐based methods for diagnosing low muscle mass and predicting mortality in patients with cirrhosis

**DOI:** 10.1111/liv.14217

**Published:** 2019-09-11

**Authors:** Rafael Paternostro, Katharina Lampichler, Constanze Bardach, Ulrika Asenbaum, Clara Landler, David Bauer, Mattias Mandorfer, Remy Schwarzer, Michael Trauner, Thomas Reiberger, Arnulf Ferlitsch

**Affiliations:** ^1^ Vienna Hepatic Hemodynamic Lab Medical University of Vienna Vienna Austria; ^2^ Divison of Gastroenterology and Hepatology Department of Internal Medicine III Medical University of Vienna Vienna Austria; ^3^ Department of Biomedical Imaging and Image‐Guided Therapy Medical University of Vienna Vienna Austria; ^4^ Department of Medicine I Hospital St. John of God Vienna Austria

**Keywords:** cirrhosis, mortality, psoas, sarcopenia, skeletal‐muscle index

## Abstract

**Background & Aims:**

Low muscle mass impacts on morbidity and mortality in cirrhosis. The skeletal‐muscle index (SMI) is a well‐validated tool to diagnose muscle wasting, but requires specialized radiologic software and expertise. Thus, we compared different Computed tomography (CT)‐based evaluation methods for muscle wasting and their prognostic value in cirrhosis.

**Methods:**

Consecutive cirrhotic patients included in a prospective registry undergoing abdominal CT scans were analysed. SMI, transversal psoas muscle thickness (TPMT), total psoas volume (TPV) and paraspinal muscle index (PSMI) were measured. Sarcopenia was defined using SMI as a reference method by applying sex‐specific cut‐offs (males: <52.4 cm^2^/m^2^; females: <38.5 cm^2^/m^2^).

**Results:**

One hundred and nine patients (71.6% male) of age 57 ± 11 years, MELD 16 (8‐26) and alcoholic liver disease (63.3%) as the main aetiology were included. According to established SMI cut‐offs, low muscle mass was present in 69 patients (63.3%) who also presented with higher MELD (17 vs 14 points; *P* = .025). The following optimal sex‐specific cut‐offs (men/women) for diagnosing low muscle mass were determined: TPMT: <10.7/ <7.8 mm/m, TPV: <194.9/ <99.2 cm^3^ and PSMI <26.3/ <20.8 cm^2^/m^2^. Thirty (27.5%) patients died during a follow‐up of 15 (0.3‐45.7) months. Univariate competing risks analyses showed a significant risk for mortality according to SMI (aSHR:2.52, 95% CI: 1.03‐6.21, *P* = .043), TPMT (aSHR: 3.87, 95% CI: 1.4‐8.09, *P* = .007) and PSMI (aSHR: 2.7, 95% CI: 1.17‐6.23, *P* = .02), but not TPV (*P* = .18) derived low muscle mass cut‐offs. In multivariate analysis only TPMT (aSHR: 2.82, 95% CI: 1.20‐6.67, *P* = .018) was associated with mortality, SMI (aSHR: 1.93, 95% CI: 0.72‐5.16, *P* = .19) and PSMI (aSHR: 1.93, 95% CI: 0.79‐4.75, *P* = .15) were not.

**Conclusion:**

Low muscle mass was highly prevalent in our cohort of patients with cirrhosis. Gender‐specific TPMT, SMI and PSMI cut‐offs for low muscle mass can help identify patients with an increased risk for mortality. Importantly, only TPMT emerged as an independent risk factor for mortality in patients with cirrhosis.

**Key points**

- Low muscle mass is a risk factor for early hepatic decompensation and death in patients with cirrhosis. Computed tomography (CT) based methods are the gold standard to diagnose low muscle mass.
- In this study we compared four different CT‐based methods for the diagnosis of low muscle mass and found the transversal psoas muscle thickness (TPMT) as an independent risk factor for mortality.
- The TPMT is easy to calculate, does not require specific radiologic software and could therefore emerge as a feasible tool for the clinical hepatologist to diagnose low muscle mass.

## 1 INTRODUCTION

Sarcopenia is highly prevalent in advanced chronic liver disease (ACLD) with reported prevalence rates ranging between 22% and 70%.^1^, ^2^ Once sarcopenia develops, prognosis is significantly impaired and patients are at increased risk for liver‐related morbidity and mortality.^1^, ^3^, ^4^, ^5^, ^6^ Based on these findings, the ‘MELD‐Sarcopenia’‐Score has been developed and has shown a higher accuracy in predicting mortality within 3 months compared to MELD alone.^7^ However, a recent study did not confirm an increase in the prognostic value of sarcopenia in addition to MELD.^4^

Computed tomography (CT)‐based methods represent the gold standard for diagnosing sarcopenia,^8^ with skeletal‐muscle index (SMI) being the most commonly used parameter. SMI is calculated from the cross‐sectional area of abdominal skeletal muscles at the third lumbar vertebrae, normalized by body height.^8^, ^9^ Although the SMI was reported to be an independent risk factor for mortality,^5^, ^10^ it has two main limitations: firstly, a specific software is needed to measure the cross‐sectional area of abdominal skeletal muscle, and secondly, this measurement requires the expertise of an experienced radiologist.

Hence, several studies have used different CT‐based methods to investigate the prognostic value of sarcopenia in cirrhosis. The total psoas muscle area and the corresponding psoas muscle‐index have been widely studied^1^ but also require volumetry, and thus, specific software. Total psoas muscle volume (TPV) predicted post‐operative complications following hepatic surgery.^11^ More recently, the paraspinal muscle index (PSMI) has been shown to predict mortality in a large cohort of patients with cirrhosis.^12^ Transversal psoas muscle thickness (TPMT) is an easy‐to‐use and readily available parameter in clinical practice since it is just based on the psoas diameter. Importantly, the TPMT was an independent risk factor for mortality on top of MELD or MELD‐Na.^3^ The detrimental impact of sarcopenia on survival has subsequently been confirmed by several studies.^4^, ^6^, ^7^, ^13^, ^14^ Thus, strategies to improve muscle wasting are now widely accepted as important treatment goals in ACLD patients,^9^, ^15^, ^16^ however standardized and clinically feasible assessment of muscle mass is still lacking and randomized controlled trials on specific interventions are rare. Intramuscular testosterone injection has been shown to increase muscle mass in male patients with ACLD, however, no beneficial effects on liver‐related outcomes were observed.^17^

Therefore, we aimed to compare four different CT‐based methods for diagnosing low muscle mass (SMI, TPMT, TPV and PSMI) in regard to their prognostic value for liver‐related mortality.

## 2 METHODS

### 2.1 Patients

**Figure 1:**
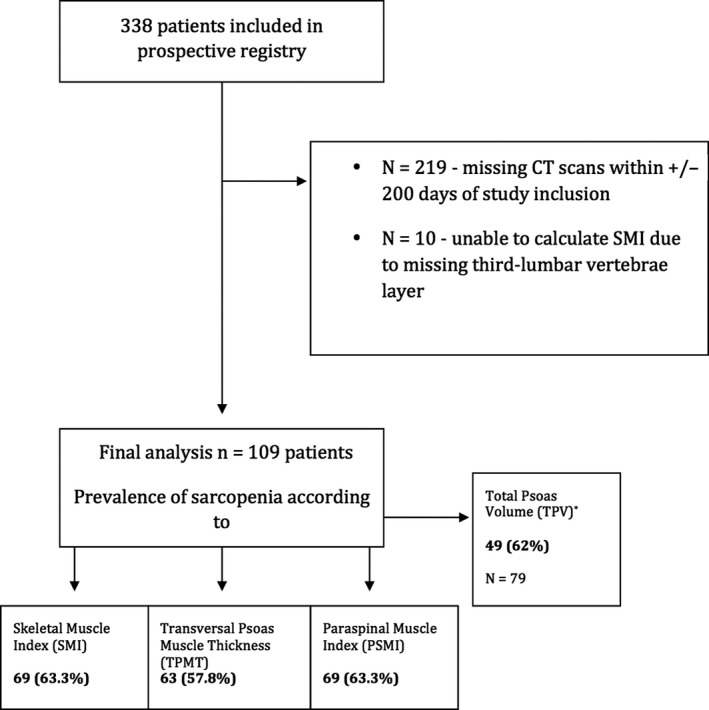
Flow‐chart and prevalence of low muscle mass according to different CT‐based methods (SMI, TPMT, PSMI and TPV)

Patients from a prospective registry undergoing a CT scan within ±200 days of inclusion were analysed (Figure 1). Inclusion criteria were: available CT scan, diagnosis of cirrhosis (based on clinical, laboratory or radiological findings)^18^ and available information on standard laboratory parameters. Exclusion criteria were: missing CT scan and/or impossibility to calculate SMI and TPMT, missing laboratory parameters or unavailability of clinical follow‐up and hepatocellular carcinoma at baseline. Inclusion of patients started in 2012, liver‐related complications (ie decompensation) were recorded and patients were followed until transplantation, death or date of last clinical visit. Survival time was assessed from the time of the CT scan until censoring/the first event, as defined above. During this study, patients were treated according to the guidelines in effect during the time.^18^, ^19^, ^20^

Out of 338 patients included in the prospective database 219 were excluded because of missing CT scans within ±200 days of study inclusion. Further 10 patients were excluded due impossible calculations of SMI and TPMT (incomplete abdominal CT scan with missing third lumbar vertebrae layer).

### 2.2 Image analysis

All measurements were obtained on axial CT scans of the abdomen performed on a multidetector CT scanner with a patient size‐adapted tube voltage (80‐120 kVp) an active tube current modulation. Seventy to one hundred and twenty millilitres (depending on the body weight) of iodinated contrast agents (300‐400 mg/mL Iodine concentration) was given intravenously at a peripheral vein at a flow rate of 4‐5 mL/s, followed by a saline flush of 20 mL using a power injector. All imaging data were acquired on transverse venous phase images using a soft tissue kernel (B30F), with a section thickness of 3 mm and a reconstruction interval of 2 mm.

The SMI, TPMT, PSMI and psoas volume were calculated in all patients at the level of the third lumbar vertebrae (L3) using OsiriX medical imaging software for iOs (Pixmeo, Version 7.5) and syngo.via software (Siemens Healthcare GmbH, Version VB30).

Total psoas volume (TPV) of the entire psoas muscle was calculated in 79 patients using syngo.via software (Siemens Healthcare GmbH, Version VB30). Thirty patients had only upper abdominal CT scans and measurement of TPV was therefore not possible.

Transverse venous phase images of the abdomen were loaded. As previously described,^21^ the third lumbar vertebral body, where both transverse processes were depictable, was identified.

The SMI was defined as the total cross‐sectional area of all abdominal muscles at the level of L3 on a single scan image normalized by height: semi‐automated demarcation of the muscle tissue was based on Hounsfield unit (HU) thresholds from −29 to +150 with manual correction by the reader. The included muscles are the psoas muscle, erector spinae, quadratus lumborum, transversus abdominis, external and internal obliques and rectus abdominis. The calculated area (=total muscle area = TMA), corrected by height, contributes to the final SMI formula, calculated as followed:

$$\text{Skeletal Muscle Index}\;\left(\text{SMI}\right)=\frac{\left(\text{Total Muscle Area}\left[{\text{cm}}^{2}\right]\right)}{\left(\text{Height}\left[m\right]\times \text{Height}\left[m\right]\right)}$$

TPMT‐L3 was defined as the transversal diameter of the right psoas muscle perpendicular to the largest axial psoas muscle diameter at the L3 endplate. The results were normalized to body height and shown as mm/m.

TPMT‐umbilical was defined as the transversal diameter of the right psoas muscle perpendicular to the largest axial psoas muscle diameter at the level of the umbilicus. Results were normalized to body height and shown as mm/m. In 11 patients TPMT‐umbilical could not measured because of missing umbilicus layers as a result of either CT scans limited to the splenoportal axis or massive ascites.

Paraspinal muscle index was defined as the bilateral, total paraspinal muscle area (psoas major and minor muscles, quadratus lumborum muscles, transversospinal muscles and erector spinae muscles) at the L3 endplate and results were normalized by height and are shown in cm^2^/m^2^.

Total psoas volume of the right psoas muscle was calculated semi‐automatically, by manual outlining of the boarders of the muscle, starting at the level of the last thoracic or first lumbar vertebra continuing until the psoas muscle becomes indistinguishable from the iliopsoas muscle. Results are shown in cm^3^.

An independent reader, instructed by a senior board‐certified radiologist analysed all variables. In case of SMI and TPMT a second independent reader, instructed by a senior board‐certified radiologist additionally analysed the variables, and mean values of both measurements where then taken into account for statistical analysis. Interrater‐variability kappa statistics was 0.70 for TPMT‐L3 and 0.75 for SMI, indicating per definition ‘substantial agreement’.

### 2.3 Definition of cut‐offs for low mucle mass

Low muscle mass was defined according to established SMI cut‐offs: males:<52.4 cm^2^/m^2^, females:<38.5 cm^2^/m^2^.^5^, ^8^, ^10^ Using SMI as the reference method AUROC analyses were done for TPMT, PSMI and TPV and the best cut‐off values to diagnose muscle wasting were determined using Youden Index. Cut‐offs for low muscle mass using SMI as the reference method:

- TPMT: male: <10.7 mm/m, female: <7.8 mm/m
- PSMI: male: <26.3 cm^2^/m^2^, female: <20.8 cm^2^/m^2^
- TPV: male: <194.9 cm^3^, female: <99.2 cm^3^

### 2.4 Statistical analysis

Continuous variables were reported as mean ± SD or median (95% confidence interval, CI) and categorical variables were reported as number (n) of patients with certain characteristic (proportion of patients with certain characteristics, %). Spearman's rho correlation coefficients were calculated to assess correlations between two variables where at least one was: distributed non‐parametric and/or categorized as an ordinal variable. Pearsons ‘R’ coefficient was used when assuming a correlation of two normally distributed quantitative variables. Student's *t* test was used for group comparisons of normally distributed data, while Mann‐Whitney‐*U* test was used where data were not normally distributed. Kruskal‐Wallis one‐way analysis of variance was used to compare medians between three or more groups. Chi‐squared test or Fisher's exact test was performed to compare differences in proportions between groups. Youden index was used to determine optimal cut‐offs for diagnosing low muscle mass (as predefined by established SMI cut‐offs) for TPMT, PSMI and TPV. Secondly, Youden Index was also used to determine optimal cut‐offs for estimating survival for all parameters: SMI, TPMT, PSMI and TPV. Differences in transplant‐free survival times between the groups that were stratified according to the previously determined SMI, TPMT, PSMI and TPV cut‐offs were assessed using log‐rank test. To investigate the effect of muscle wasting (as defined by SMI, TPMT, PSMI and TPV) on survival, considering liver transplantation as a competing risk, we used Fine and Gray competing risks regression models (cmprsk: Subdistribution Analysis of Competing Risks; https://CRAN.R-project.org/package=cmprsk).

The low muscle mass cut‐offs for SMI, TPMT, PSMI and TPV were separately tested in uni‐ and multivariate. Variables that showed difference in univariate analysis and those considered clinically highly relevant were included in the multivariate analysis. Two‐sided *P* < .05 were considered as statistically significant. IBM SPSS 24.0 (SPSS Inc) and R Core Team (2019; R: A language and environment for statistical computing. R Foundation for Statistical Computing, Vienna, Austria. URL https://www.R-project.org/) was used for statistical analyses.

### 2.5 Ethics

This study was approved by the ethics committee of the Medical University of Vienna (No. 1584/2012) and performed in accordance with the Declaration of Helsinki. All patients signed a written informed consent for study inclusion.

## 3 RESULTS

### 3.1 Patient population

One hundred and nine patients were included in this study. Low muscle mass as defined using SMI was present in 69 patients (63.3%; Table 1). The main patient characteristics stratified according to presence or absence of low muscle mass are summarized in Table 1. Thirty patients (27.5%) died during follow‐up, significantly more among those with low muscle mass (66.7% vs 33.3%, *P* = .049). Reasons of death were: Acute on chronic liver failure 19 (63.3%) [decompensated cirrhosis and multi‐organ failure n = 12 (63%), sepsis/infection n = 6 (32%), haemorrhagic shock n = 1 (5%)], hepatocellular carcinoma n = 5 (16.6%), unknown n = 4 (13.4%) and cardiac decompensation 2 (6.7%). Twenty (18.4%) patients were transplanted during follow‐up.

**Table 1:** Main patients characteristics according to presence of low muscle mass defined by SMI categories (Women <38,5 cm^2^/m^2^; Men <52,4 cm^2^/m^2^)

	Normal muscle mass(n = 40)	Low muscle mass (n = 69)	*P*‐value
Age, mean ± SD	56 ± 10.7	58 ± 10.5	.462
Weight, mean ± SD	81.8 ± 17.7	80.7 ± 15.1	.746
Height, mean ± SD	1.68 ± 0.08	1.74 ± 0.77	**<.001**
BMI, median (95% CI)	28.5 (21.8‐41.7)	25.8 (20.1‐35.1)	**.009**
Gender, n(%)			
Male	20 (50%)	58 (84.1%)	**<.001**
Female	20 (50%)	11 (15.9%)	
Aetiology, n(%)			
ALD	22 (31.9%)	47 (68.1%)	.654
Viral	6 (42.9%)	8 (57.1%)	
Metabolic	3 (60%)	2 (40%)	
Cholestatic	2 (40%)	3 (60%)	
Other	7 (43.8%)	9 (56.3%)	
MELD Score, median (95% CI)	13.65 (7.5‐25)	17.15 (7.9‐28.4)	**.025**
MELD, median (95% CI)			
<16	25 (47.2%)	28 (52.8%)	**.028**
≥16	15 (26.8%)	41 (73.2%)	
Ascites, n (%)			
Grade 1	12 (50%)	12 (50%)	.189
Grade 2	21 (30.4%)	48 (69.6%)	
Grade 3	7 (43.8%)	9 (56.3%)	
Hepatic encephalopathy, n (%)			
No	28 (70%)	45 (65.2%)	.609
Yes	12 (30%)	24 (34.8%)	
NH3, mmol/L	49.65 (19.3‐100)	44.5 (20.1‐126.48)	.932
Creatinine, mg/dL	0.82 (0.62‐1.57)	0.95 (0.49‐2.53)	.354
Sodium, mmol/L	137 (127‐145)	134 (126‐143)	.178
Bilirubin, mg/dL	1.53 (0.32‐5.2)	1.95 (0.52‐10.56)	**.031**
Platelets, G/L	92 (32‐317)	130 (35‐271)	**<.001**
INR	1.3 (1.1‐1.5)	1.3 (1.2‐1.5)	.523
C‐reactive Protein, mg/dL	0.39 (0.09‐4.12)	1.16 (0.05‐6.48)	**.008**
Albumin, g/L	35.6 ± 6.9	33.2 ± 6.3	.070
PSMI, cm^2^/m^2^	26.5 ± 5	21.9 ± 3.6	**<.001**
TPV, cm^3^	166 ± 56.4	150.9 ± 47.2	.208
TPMT, mm/m	10.8 ± 3.1	8.8 ± 2.5	**<.001**
Death, n (%)			
No	34 (85%)	45 (65.2%)	**.026**
Yes	6 (15%)	24 (34.8%)	

Abbreviations: PSMI, paraspinal muscle index; SMI, skeletal‐muscle index; TPMT, transversal psoas muscle thickness; TPV, total psoas volume. Bold indicates the significant *p*‐value.

### 3.2 Establishing low muscle mass cut‐offs in cirrhosis for TPMT, PSMI and TPV

Using SMI as the reference method to define muscle wasting, diagnostic cut‐offs for low muscle mass were calculated for the other CT‐based muscle mass indices as described above. TPMT (AUC: 0.70, 95% CI: 0.59‐0.81; *P* = .001) and PSMI (AUC: 0.77, 95% CI: 0.67‐0.86; *P* < .001) had significant diagnostic accuracy for detecting (SMI‐defined) low muscle mass. However, TPV (AUC: 0.56; 95% CI: 0.42‐0.70; *P* = .392) did not reflect (SMI‐defined) muscle wasting in our cohort of patients with cirrhosis. AUROC curves are shown in Figure S1. Sixty three (57.8%), 69 (63.3%) and 49 (62%) were diagnosed with low muscle mass according to SMI‐derived TPMT, PSMI and TPV cut‐offs (Figure 1).

### 3.3 Low muscle mass—as defined using SMI vs TPMT vs PSMI vs TPV to predict mortality

**Figure 2:**
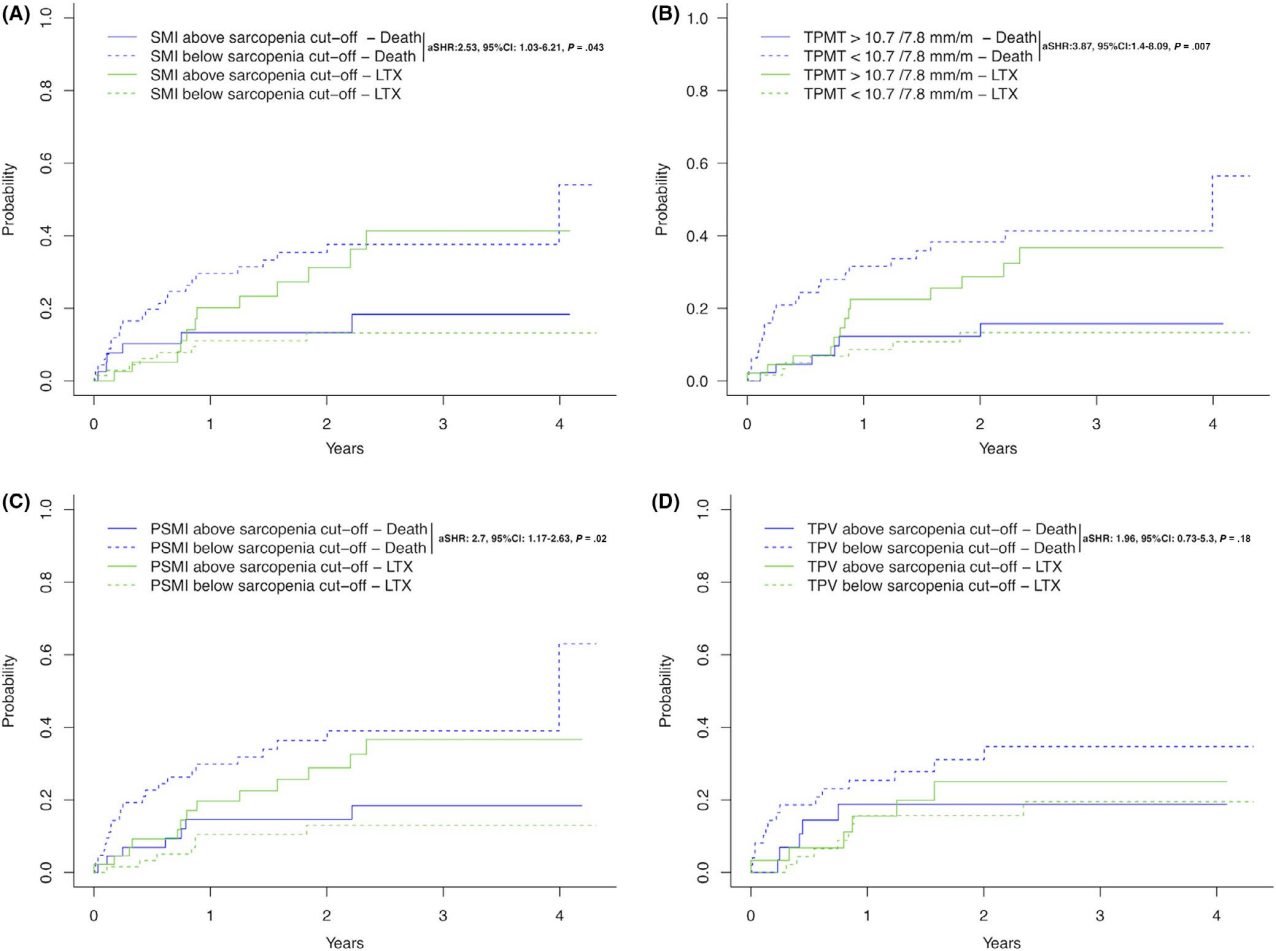
Competing risks analysis (event of interest: death, competing risks: liver transplantation) for musclemass‐derived cut‐offs as defined using SMI (Panel A), TPMT (Panel B), PSMI (Panel C) and TPV (Panel D)

To compare the predictive value of each CT‐based definition method we performed competing risks analyses for each definition (Figure 2A‐D). Univariate competing risks analyses found a significant risk for mortality when patients were stratified according to SMI‐ (aSHR: 2.52, 95% CI: 1.03‐6.21, *P* = .043, Figure 2A), TPMT‐ (aSHR: 3.87, 95% CI: 1.4‐8.09, *P* = .007, Figure 2B) and PSMI‐ (aSHR: 2.7, 95% CI: 1.17‐6.23, *P* = .02, Figure 2C) derived low muscle mass cut‐offs (Table S1).

In contrast TPV‐defined low muscle mass (aSHR: 1.96, 95% CI: 0.73‐5.3, *P* = .18, Figure 2D) was not associated with an increased risk for mortality.

Subsequently, we evaluated the independent predictive value of SMI‐, TPMT‐ and PSMI‐defined low muscle mass using multivariate competing risks models (Model‐1: SMI, Model‐2: TPMT; Model 3: PSMI; Table S1).

Interestingly, only TPMT (aSHR: 2.82, 95% CI: 1.20‐6.67, *P* = .018) was independently associated with mortality in our competing risks model, while SMI (aSHR: 1.93, 95% CI: 0.72‐5.16, *P* = .19) and PSMI (aSHR: 1.93, 95% CI: 0.79‐4.75, *P* = .15) were not.

### 3.4 Defining cut‐offs for muscle mass parameters that correlate with survival

Using mortality as the endpoint, cut‐offs were calculated based on AUROC analysis and Youden index. The SMI‐AUC was 0.63 (0.51‐0.74, *P* = .04), TPMT‐AUC: 0.61 (0.51‐0.72, *P* = .069), PSMI‐AUC: 0.59 (0.47‐0.71, *P* = .061) and TPV‐AUC: 0.54 (0.40‐0.69, *P* = .565). Cut‐offs for mortality were:

- SMI: male: <42.9 cm^2^/m^2^; female: <36.3 cm^2^/m^2^
- TPMT: male: <12 mm/m; female: <8 mm/m
- PSMI: male: <25.3 cm^2^/m^2^; female: <18.9 cm^2^/m^2^
- TPV: male: <144.5 cm^3^; female: <128.4 cm^3^

### 3.5 Prediction of mortality by muscle mass parameters (survival cut‐offs)

**Figure 3:**
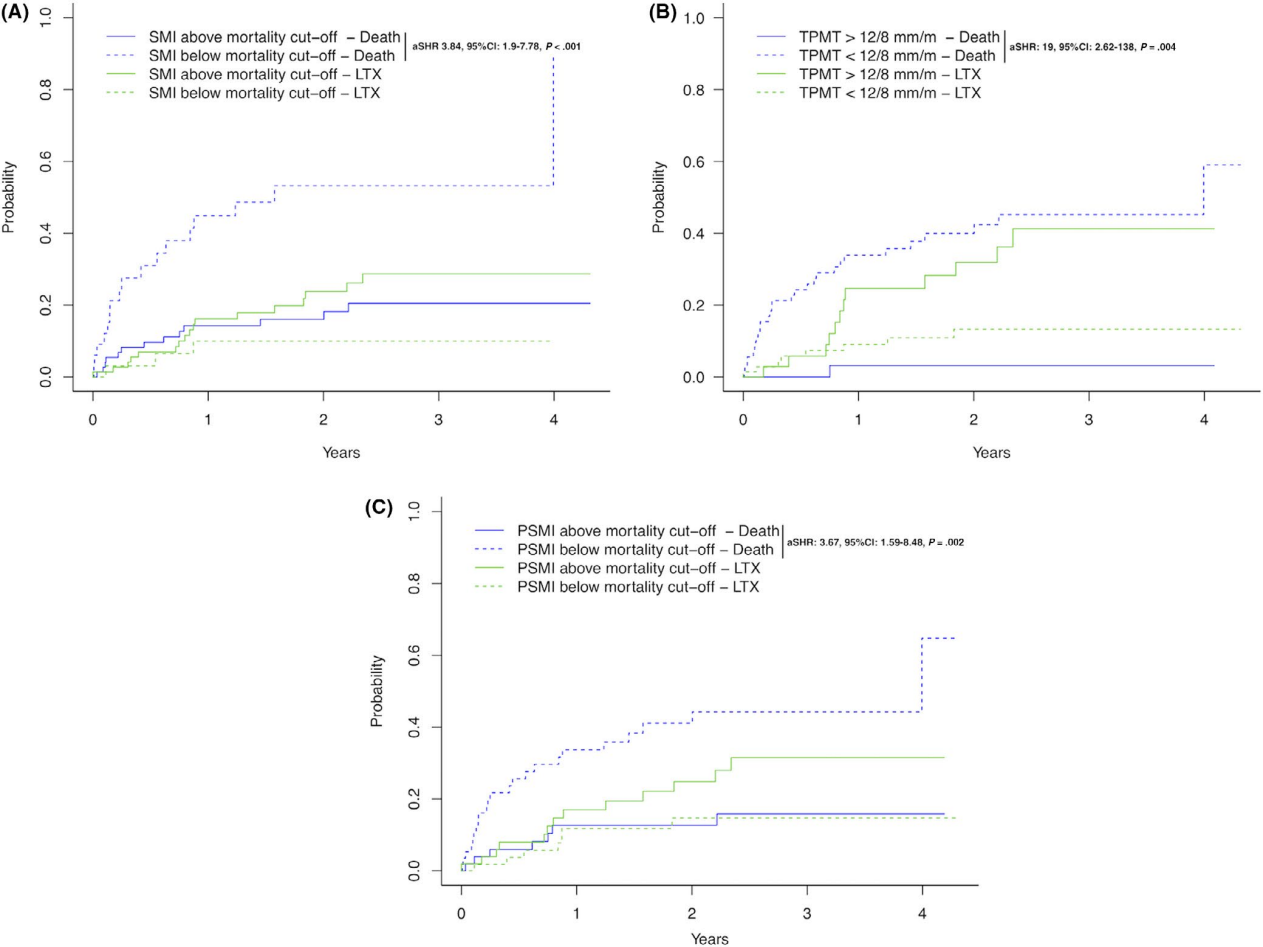
Competing risks analysis (event of interest: death, competing risks: liver transplantation) for mortality‐derived cut‐offs for SMI (Panel A), TPMT (Panel B) and SMI (Panel C)

Univariate competing risks analysis showed a significantly higher risk for mortality when SMI‐ (aSHR: 3.84, 95% CI: 1.9‐7.78, *P* < .001; Figure 3A), TPMT‐ (aSHR: 19, 95% CI: 2.62‐138, *P* = .004; Figure 3B), PSMI‐ (aSHR: 3.67, 95% CI: 1.59‐8.48, *P* = .002, Figure 3C) but not TPV‐ (aSHR:1.74, 95% CI: 0.73‐4.17, *P* = .21) derived mortality cut‐offs were used.

We then performed multivariate competing risks analysis and found SMI‐ (aSHR: 2.96, 95% CI: 1.41‐6.21, *P* = .004, Table 2) TPMT‐ (aSHR: 16.18, 95% CI: 2.15‐122.04, *P* = .004, Table 2) and PSMI‐ (aSHR: 2.64, 95% CI: 1.08‐6.43, *P* = .03, Table 2) derived mortality cut‐offs independently associated with mortality.

**Table 2:** Competing risks analyses (event of interest: mortality, competing risk: liver transplantation) adjusted for age, MELD, albumin, presence of ascites and considering for mortality‐defined cut‐offs for either SMI (Model 1) or TPMT (Model 2) or PSMI (Model 3)

Variables	Univariate			MV‐Model 1 SMI			MV‐Model 2 TPMT			MV‐Model 3 PSMI		
	aSHR	95% CI	*P*‐value	aSHR	95% CI	*P*‐value	aSHR	95% CI	*P*‐value	aSHR	95% CI	*P*‐value
Age, per year	1.04	0.99‐1.08	.12	1.03	0.98‐1.07	.23	1.04	0.99‐1.08	.11	1.03	0.98‐1.07	.23
MELD, per point	1.09	1.02‐1.17	.009	1.03	0.94‐1.13	.57	1.05	0.96‐1.15	.25	1.05	0.96‐1.15	.27
Albumin, per unit (mg/L)	0.93	0.88‐0.97	.002	0.95	0.88‐1.02	.13	0.96	0.89‐1.03	.25	0.96	0.90‐1.04	.33
Ascites, yes vs no	2.28	0.84‐6.17	.10	1.24	0.43‐3.60	.69	0.93	0.37‐2.35	.88	1.22	0.39‐3.88	.73
SMI cut‐off	3.84	1.9‐7.78	<.001	*2.96*	*1.41‐6.21*	*.004*	—	—	—	—	—	—
TPMT cut‐off	19	2.62‐138	.004	—	—	—	*16.18*	*2.15‐122.04*	*.007*	—	—	—
PSMI cut‐off	3.67	1.59‐8.48	.002	—	—	—	—	—	—	*2.64*	*1.08‐6.43*	*.03*
TPV cut‐off	1.74	0.73‐4.17	.21	—	—	—	—	—	—	—	—	—

Abbreviations: PSMI, paraspinal muscle index; SMI, skeletal‐muscle index; TPMT, transversal psoas muscle thickness; TPV, total psoas volume. Italics indicates significant *p*‐values in the multivariate analyses.

## 4 DISCUSSION

Giving the detrimental effects of sarcopenia on liver‐related outcomes and mortality in patients with ACLD, reversing muscle‐loss represents a management priority in these patients.^9^, ^22^ Sarcopenia in ACLD is multifactorial and the pathophysiology include protein‐malnutrition, increased proteolysis from skeletal muscles, accelerated starvation response, physical inactivity or humoral factors such as upregulation of myostatin as a result of hyperammonaemia or hypotestosteronism.^8^, ^9^, ^22^, ^23^, ^24^ Nutritional supplementation with branched chain amino acids (BCAAs) has been shown to improve liver‐related outcomes^25^ and muscle mass.^26^ Furthermore exercise interventions have resulted in improvement of cardiorespiratory endurance,^27^, ^28^ muscle mass^27^ and a reduction in hepatic venous pressure gradient.^29^, ^30^ More recently intramuscular testosterone supplementation has been shown to improve muscle mass, although no effect on liver‐related outcomes was observed.^17^ Furthermore transjugular intrahepatic portosystemic shunt (TIPS)‐implantation has been shown to potentially reverse sarcopenia^31^ and this could be an important early intervention in sarcopenic patients with refractory ascites^32^ or variceal bleeding.^33^ Nevertheless, the only available data that show an improved survival when overcoming sarcopenia come from trials investigating patients before and after TIPS.^31^, ^34^ While persistence of sarcopenia after TIPS was associated with an impaired outcome, it is unclear whether this is of associative (since patients with sarcopenia pre‐TIPS typically present with more severe liver disease) or causative reasons.^31^, ^34^, ^35^ A variety of methods to measure thickness or volume of the psoas major muscle on CT scans have been published^1^, ^8^ and a recent study 36 that compared the psoas muscle area (PMA) to the SMI showed that PMA outperformed SMI in the prediction of 1‐year mortality after liver transplantation.

This is the first study comparing four CT‐based methods for the diagnosis of low muscle mass, in regard to their diagnostic and prognostic value in patients with ACLD.

Most studies evaluating muscle mass in cirrhosis have used the SMI as the reference method and used SMI cut‐offs derived from an oncology‐cohort published by Prado et al.^21^ Using SMI, several studies^4^, ^5^, ^7^, ^10^, ^37^, ^38^ have demonstrated that low muscle mass is associated with impaired survival in patients with cirrhosis. More recently, one study investigated cirrhosis‐specific SMI cut‐offs^37^ and proposed new SMI‐derived cut‐offs for the diagnosis of sarcopenia in ACLD: for men 50 cm^2^/m^2^ and for women: 30 cm^2^/m^2^), and recently the European Association for the Study of the Liver (EASL) suggested cut‐offs of <50/ <39 cm^2^.^39^ In our cohort applying the EASL proposed cut‐offs would have reclassified five patients, nevertheless the predictive value for mortality remained the same (Figure S3). Using SMI as the reference method,^21^ the prevalence of low muscle mass was as high as 63% in our ACLD cohort. Similarly high rates of muscle wasting in cirrhosis have previously been reported^5^, ^10^ in other studies and suggested that sarcopenia increases the risk for decompensation and death.^3^, ^5^, ^10^

In a recent review Kim et al^1^ reported on 10 studies using SMI, eight studies using total psoas muscle area and two studies using bioelectrical impedance for defining sarcopenia. This shows the wide spectrum and vast amount of data that are already available while new diagnostic methods are continuously being reported, such as the PSMI which independently predicted mortality in cirrhosis.^12^ Additionally, sarcopenia as diagnosed via TPV has been shown to predict post‐surgical complications in patients with hepatic malignancies.^11^

One main limitation of the SMI as the reference method, is the complexity of the method to measure the cross‐sectional abdominal muscle area that requires time and certain radiological expertise and training as well as specialized software. This largely impacts on the feasibility of calculating SMI in daily clinical practice.

In 2014, Durand et al^3^ showed that the TPMT predicts mortality independently of MELD and MELD‐Na. The major advantage of calculating TPMT values is (as compared to SMI) its accessibility. If an abdominal CT scan is available (eg due to HCC screening) the diameter perpendicular to the largest axial psoas muscle thickness can easily be measured at the endplate of the third lumbar vertebrae, corresponding to the transversal psoas thickness (TPMT). Recently, Huguet et al^40^ found excellent interobserver agreement for TPMT measurement when readings are performed by an experienced and native operator. The prognostic value of TPMT was already shown in patients with ascites^13^ and TPMT was also associated with mortality in patients on the liver transplant waiting list. Gu et al^14^ compared SMI and TPMT measurements and found good correlation between the two variables and described similar rates for sarcopenia when using SMI (37%) and TPMT (between 35% and 44%) when using gender‐specific cut‐offs for men (17.3 mm/m) and for women (10.4 mm/m).^14^

In our study, we could show that low muscle mass in ACLD can be identified using different CT‐based diagnostic algorithms, thereby we found a high prevalence of low muscle mass in up to two thirds of ACLD patients. Importantly, lower mortality‐derived muscle mass cut‐offs for SMI and similar ones for TPMT and PSMI were found to already be associated with increased mortality in our ACLD cohort. Most strikingly, the TPMT was the only independent predictor of mortality, irrespective of the chosen cut‐off (muscle mass‐ or mortality‐derived), on multivariate analyses and therefore even outperforming the SMI that requires much more infrastructure and resources to obtain. Whether measuring the TPMT at the level of the umbilicus or the third lumbar vertebrae as the method of choice is debated. Recently, Praktiknjo et al^34^, ^41^ described very heterogenous locations of the umbilicus in their study (L4 in 70%, L5 in 20% and L3 in 10%), which may add substantial variability to the measurement of TPMT that is related to the measurement position, rather than psoas muscle itself. We also measured TPMT at the umbilicus level and also observed considerable heterogeneity regarding the relation between the axial skeleton and the umbilicus and were not able to measure TPMT‐umbilical in 11 patients because of missing umbilicus on the CT scan (mostly because of CT scans limited to the splenoportal axis and massive ascites). Nevertheless we found a good prognostic capability of gender specific, mortality‐derived TPMT‐umbilical cut‐offs in the 98 remaining patients (Figure S2).Thus, we propose to use gender‐specific mortality‐derived TPMT‐L3 cut‐offs for men and women at <12 mm/m and at <8 mm/m respectively, to identify low muscle mass already at a level that impacts on mortality in ACLD.

The limitations of the PSMI as well include the requirement of specific software and expertise for its calculation which impacts on feasibility in daily clinical practice. TPV did not show prognostic significance and the requirement of scanning of the whole psoas major muscle would need special attention when designing abdominal CT protocols. Limitations of our study include the sample size and lack of longitudinal assessment of muscle mass. Another limitation is that CT scans within 200 days of study inclusion were considered. We chose this cut‐off since in daily clinical practice cirrhotic patients are usually seen every 3‐6 months in the outpatient clinic. Nevertheless, the median time of CT to study inclusion in our cohort was only 4 (−35‐36) days and therefore within a very close time to study inclusion. Arguably, the TPMT cut‐offs proposed in our study are lower than those reported by Durand et al,^3^ which can easily be explained by the anatomical structure of the psoas major muscle: When descending from its proximal origin at the level of T12‐L1 to the distal origin (L5‐S1)^42^, ^43^ the diameter increases. Therefore, TPMT values are lower when measuring at more proximal levels. Nevertheless, the median TPMT at the umbilical level in our cohort was 17.7 mm/m (14.5‐20.5), and therefore quite comparable to Durand et al.^3^ Interestingly we did not see a difference in the prevalence of presence of hepatic encephalopathy (HE) between patients with “normal” vs. low muscle‐mass, even though hyperammonemia can lead to muscle wasting.^22^ This could be explained by the fact that the presence or absence of HE was classified according to clinical presentation of the patient, not ammonia levels. Furthermore ammonia levels do not correlate with the severity of hepatic encephalopathy.^44^, ^45^, ^46^ Another possible explanation might be the link between muscle wasting and already minimal hepatic encephalopathy (MHE) as suggested by a recent study.^47^ MHE was at the time not generally evaluated in our cohort, therefore we might have under‐diagnosed MHE and this could represent the possible bias why we found no link between HE and muscle wasting. Still, our study population represents a well‐characterized consecutive cohort of ACLD patients recruited in to prospective registry. Furthermore future studies should assess if TPMT measurements by hepatologist are of similar prognostic value as those made by trained radiologist. Ultimately this could lead to an easy and rapid ‘bed‐side’ diagnosis of muscle wasting in daily clinical practice.

In conclusion, low muscle mass is highly prevalent in cirrhosis. The TPMT is a valuable tool to diagnose muscle wasting and to identify patients at risk for increased mortality. The advantages of the TPMT are its high accessibility and feasibility and this might enable clinicians to diagnose low muscle mass in daily clinical practice. Ultimately, timely diagnosis of low muscle mass at gender‐specific TPMT cut‐offs might accelerate the initiation of specific treatment strategies against muscle wasting in ACLD patients.

## CONFLICT OF INTEREST

The authors report no real or potential conflict of interest related to this study. The following conflicts of interests outside this study exist: RP, KL, CB, UA, CL, DB and RS report no conflict of interests. MM Speaker and/or consultant and/or advisory board member for AbbVie, Bristol‐Myers Squibb, Gilead, W. L. Gore & Associates and Janssen. MT: Speaker for BMS, Falk Foundation, Gilead and MSD; advisory boards for Albireo, Falk Pharma GmbH, Genfit, Gilead, Intercept, MSD, Novartis, Phenex and Regulus. He further received travel grants from Abbvie, Falk, Gilead and Intercept and unrestricted research grants from Albireo, Cymabay, Falk, Gilead, Intercept, MSD and Takeda. He is also the coinventor of patents on the medical use of norUDCA filed by the Medical University of Graz. TR: Grant support from Abbvie, Boehringer‐Ingelheim, Gilead, MSD, Philips Healthcare, Gore; speaking honoraria from Gilead, Gore, Intercept, Roche, MSD; consulting/advisory board fee from Abbvie, Boehringer‐Ingelheim, Gilead, MSD; and travel support from Boehringer‐Ingelheim, Gilead and Roche. AF: Speaker and/or consultant and/or advisory board member for AbbVie, Gilead and Intercept. AF owns a patent on a catheter for the measurement of hepatic venous pressure gradient.

## AUTHOR CONTRIBUTIONS

All authors contributed either to the research design (RP, AF), and/or the acquisition (clinical data: RP, CL, MM, RS, TR, radiological data: KL, CB, UA) analysis (RP, AF, DB TR, MM) or interpretation (all authors) of data. RP drafted the manuscript, which was then critically revised by all other authors. All authors approved the final version of this manuscript.

## APPENDIX 1

**Table A1:** STROBE Statement—checklist of items that should be included in reports of observational studies

	**Item No**	**Recommendation**	**Page No**
Title and abstract	1	(a) Indicate the study's design with a commonly used term in the title or the abstract	3
		(b) Provide in the abstract an informative and balanced summary of what was done and what was found	3
Introduction			
Background/rationale	2	Explain the scientific background and rationale for the investigation being reported	4‐5
Objectives	3	State specific objectives, including any prespecified hypotheses	4‐5
Methods			
Study design	4	Present key elements of study design early in the paper	5‐7
Setting	5	Describe the setting, locations and relevant dates, including periods of recruitment, exposure, follow‐up and data collection	5‐7
Participants	6	(a) *Cohort study*—Give the eligibility criteria, and the sources and methods of selection of participants. Describe methods of follow‐up *Case‐control study*—Give the eligibility criteria, and the sources and methods of case ascertainment and control selection. Give the rationale for the choice of cases and controls *Cross‐sectional study*—Give the eligibility criteria, and the sources and methods of selection of participants	5‐7
		(b) *Cohort study*—For matched studies, give matching criteria and number of exposed and unexposed *Case‐control study*—For matched studies, give matching criteria and the number of controls per case	n.a.
Variables	7	Clearly define all outcomes, exposures, predictors, potential confounders and effect modifiers. Give diagnostic criteria, if applicable	5‐7
Data sources/ measurement	8^a^	For each variable of interest, give sources of data and details of methods of assessment (measurement). Describe comparability of assessment methods if there is more than one group	5‐7
Bias	9	Describe any efforts to address potential sources of bias	5‐7
Study size	10	Explain how the study size was arrived at	5‐7
Quantitative variables	11	Explain how quantitative variables were handled in the analyses. If applicable, describe which groupings were chosen and why	7‐8
Statistical methods	12	(a) Describe all statistical methods, including those used to control for confounding	7‐8
		(b) Describe any methods used to examine subgroups and interactions	7‐8
		(c) Explain how missing data were addressed	7‐8
		(d) *Cohort study*—If applicable, explain how loss to follow‐up was addressed *Case‐control study*—If applicable, explain how matching of cases and controls was addressed *Cross‐sectional study*—If applicable, describe analytical methods taking account of sampling strategy	7‐8
		(e) Describe any sensitivity analyses	7‐8
Results			
Participants	13^a^	(a) Report numbers of individuals at each stage of study—eg numbers potentially eligible, examined for eligibility, confirmed eligible, included in the study, completing follow‐up and analysed	5 and 18
		(b) Give reasons for non‐participation at each stage	5 and 18
		(c) Consider use of a flow diagram	18
Descriptive data	14^a^	(a) Give characteristics of study participants (eg demographic, clinical, social) and information on exposures and potential confounders	8‐9 and 16
		(b) Indicate number of participants with missing data for each variable of interest	8‐9 and 16
		(c) *Cohort study*—Summarize follow‐up time (eg average and total amount)	8‐9 and 16
Outcome data	15^a^	*Cohort study*—Report numbers of outcome events or summary measures over time	8‐9 and 16
		*Case‐control study—*Report numbers in each exposure category, or summary measures of exposure	
		*Cross‐sectional study—*Report numbers of outcome events or summary measures	
Main results	16	(a) Give unadjusted estimates and, if applicable, confounder‐adjusted estimates and their precision (eg 95% confidence interval). Make clear which confounders were adjusted for and why they were included	8‐9 and 17
		(b) Report category boundaries when continuous variables were categorized	8‐9
		(c) If relevant, consider translating estimates of relative risk into absolute risk for a meaningful time period	n.a.
Other analyses	17	Report other analyses done—eg analyses of subgroups and interactions, and sensitivity analyses	8‐9 and 16 and Suppl.
Discussion			
Key results	18	Summarize key results with reference to study objectives	10‐12
Limitations	19	Discuss limitations of the study, taking into account sources of potential bias or imprecision. Discuss both direction and magnitude of any potential bias	10‐12
Interpretation	20	Give a cautious overall interpretation of results considering objectives, limitations, multiplicity of analyses, results from similar studies and other relevant evidence	10‐12
Generalizability	21	Discuss the generalizability (external validity) of the study results	10‐12
Other information			
Funding	22	Give the source of funding and the role of the funders for the present study and, if applicable, for the original study on which the present article is based	1

Note

An Explanation and Elaboration article discusses each checklist item and gives methodological background and published examples of transparent reporting. The STROBE checklist is best used in conjunction with this article (freely available on the Web sites of PLoS Medicine at http://www.plosmedicine.org/, Annals of Internal Medicine at http://www.annals.org/, and Epidemiology at http://www.epidem.com/). Information on the STROBE Initiative is available at http://www.strobe-statement.org.

a Give information separately for cases and controls in case‐control studies and, if applicable, for exposed and unexposed groups in cohort and cross‐sectional studies.
