# Supplementary material for: The value of different CT‐based methods for diagnosing low muscle mass and predicting mortality in patients with cirrhosis
Source: Liver Int. 2019 Sep 11;39(12):2374–85. doi: 10.1111/liv.14217 (PMC6899596; doi:10.1111/liv.14217)
Supplement: Supplementary file 1 [file LIV-39-2374-s001.docx]

**Supplemental Table 1:**

Competing risks analysis (event of interest: death, competing risk: liver transplantation) adusted for age, MELD, albumin, presence of ascites and **muscle wasting defining cut-offs** for either SMI (Model 1) , TPMT (Model 2), PSMI (Model 3) or TPV.

|  | **Univariate** | | | **MV-Model 1**  **SMI** | | | **MV-Model 2**  **TPMT** | | | **MV-Model 3**  **PSMI** | | |
| --- | --- | --- | --- | --- | --- | --- | --- | --- | --- | --- | --- | --- |
| **Variables** | **aSHR** | **95%CI** | **p-value** | **aSHR** | **95%CI** | **p-value** | **aSHR** | **95%CI** | **p-value** | **aSHR** | **95%CI** | **p-value** |
| **Age, per year** | 1.04 | 0.99-1.08 | 0.12 | 1.04 | 0.99-1.08 | 0.099 | 1.03 | 0.99-1.08 | 0.16 | 1.03 | 0.99-1.09 | 0.15 |
| **MELD, per point** | 1.09 | 1.02-1.17 | 0.009 | 1.06 | 0.96-1.16 | 0.260 | 1.05 | 0.96-1.15 | 0.28 | 1.05 | 0.96-1.16 | 0.27 |
| **Albumin, per unit (mg/l)** | 0.93 | 0.88-0.97 | 0.002 | 0.96 | 0.89-1.03 | 0.270 | 0.95 | 0.89-1.02 | 0.14 | 0.96 | 0.89-1.03 | 0.26 |
| **Ascites, yes vs. no** | 2.28 | 0.84-6.17 | 0.10 | 1.22 | 0.38-3.93 | 0.740 | 1.10 | 0.38-3.18 | 0.86 | 1.25 | 0.39-4.02 | 0.71 |
| **SMI**  **Cut-off** | 2.53 | 1.03-6.21 | 0.043 | *1.93* | *0.72-5.16* | *0.19* | - | - | - | - | - | - |
| **TPMT Cut-off** | 3.87 | 1.4-8.09 | 0.007 | - | - | - | *2.82* | *1.20-6.67* | *0.018* | - | - | - |
| **PSMI**  **Cut-off** | 2.7 | 1.17-6.23 | 0.02 | - | - | - | - | - | - | *1.93* | *0.79-4.75* | *0.15* |
| **TPV**  **Cut-off** | 1.96 | 0.73-5.3 | 0.18 | - | - | - | - | - | - | - | - | - |

**Supplemental Table 2: Competing risks analysis of factors associated with the probability for liver transplantation during follow-up (event of interest: liver transplantation, competing risk: death).**

|  | **MV-Model 1**  **SMI** | | | **MV-Model 2**  **TPMT** | | | **MV-Model 3**  **PSMI** | | |
| --- | --- | --- | --- | --- | --- | --- | --- | --- | --- |
| **Variables** | **aSHR** | **95%CI** | **p-value** | **aSHR** | **95%CI** | **p-value** | **aSHR** | **95%CI** | **p-value** |
| **Age, per year** | 0.99 | 0.95-1.04 | 0.76 | 0.99 | 0.95-1.04 | 0.910 | 1.0 | 0.96-1.04 | 1.0 |
| **MELD, per point** | 1.09 | 1.02-1.16 | 0.008 | 1.08 | 1.002-1.16 | 0.044 | 1.09 | 1.01-1.16 | 0.021 |
| **Albumin, per unit (mg/l)** | 0.94 | 0.86-1.01 | 0.09 | 0.96 | 0.89-1.04 | 0.270 | 0.94 | 0.86-1.02 | 0.12 |
| **Ascites, yes vs. no** | 3.28 | 0.64-16.89 | 0.15 | 2.76 | 0.61-12.56 | 0.190 | 2.75 | 0.56-13.43 | 0.21 |
| **SMI**  **cut-off** | 0.18 | 0.07-0.48 | <0.001 | - | - | - | - | - | - |
| **TPMT cut off** | - | - | - | 0.25 | 0.09-0.72 | 0.010 | - | - | - |
| **PSMI**  **Cut off** | - | - | - | - | - | - | 0.20 | 0.07-0.54 | 0.002 |

**Supplemental Figure 1:** AUROC for detecting sarcopenia using SMI as the reference methods for (A) TPMT and PSMI (B) TPV (n=79).

**A**

**Figure 1B**

**Supplemental Figure 2:** Kaplan Meier survival curves shown for patients stratified according to their gender-specific mortality derived TPMT measured at the umbilicus level. Measurement was only possible in 98 patients. In 11 patients, TPMT at the umbilicus level could not be measured because of missing umbilical layers due to either CT scans limited to the splenoportal axis or grade 3 ascites.

**Supplemental Figure 3:** Competing risks analysis (event of interest: death, competing risks: liver transplantation) for SMI as defined according to the current EASL guidelines.
